# Combining multicriteria decision analysis, ethics and health technology assessment: applying the EVIDEM decisionmaking framework to growth hormone for Turner syndrome patients

**DOI:** 10.1186/1478-7547-8-4

**Published:** 2010-04-08

**Authors:** Mireille M Goetghebeur, Monika Wagner, Hanane Khoury, Donna Rindress, Jean-Pierre Grégoire, Cheri Deal

**Affiliations:** 1 BioMedCom Consultants inc, Dorval, Quebec, Canada; 2 Population Health Research Unit of the CHA, and Faculty of Pharmacy, Laval University, Québec, Canada; 3 Endocrinology Service and Research Center of the CHU Sainte-Justine, and Department of Pediatrics, University of Montréal, Montreal, Québec, Canada

## Abstract

**Objectives:**

To test and further develop a healthcare policy and clinical decision support framework using growth hormone (GH) for Turner syndrome (TS) as a complex case study.

**Methods:**

The EVIDEM framework was further developed to complement the multicriteria decision analysis (MCDA) Value Matrix, that includes 15 quantifiable components of decision clustered in four domains (quality of evidence, disease, intervention and economics), with a qualitative tool including six ethical and health system-related components of decision. An extensive review of the literature was performed to develop a health technology assessment report (HTA) tailored to each component of decision, and content was validated by experts. A panel of representative stakeholders then estimated the MCDA value of GH for TS in Canada by assigning weights and scores to each MCDA component of decision and then considered the impact of non-quantifiable components of decision.

**Results:**

Applying the framework revealed significant data gaps and the importance of aligning research questions with data needs to truly inform decision. Panelists estimated the value of GH for TS at 41% of maximum value on the MCDA scale, with good agreement at the individual level (retest value 40%; ICC: 0.687) and large variation across panelists. Main contributors to this panel specific value were "Improvement of efficacy", "Disease severity" and "Quality of evidence". Ethical considerations on utility, efficiency and fairness as well as potential misuse of GH had mixed effects on the perceived value of the treatment.

**Conclusions:**

This framework is proposed as a pragmatic step beyond the current cost-effectiveness model, combining HTA, MCDA, values and ethics. It supports systematic consideration of all components of decision and available evidence for greater transparency. Further testing and validation is needed to build up MCDA approaches combined with pragmatic HTA in healthcare decisionmaking.

## Background

Healthcare decisionmaking is a complex process requiring simultaneous consideration of a number of elements including scientific judgment, economics and ethics. The cost-effectiveness (CE) model has become a prime model for healthcare resource allocation and decisionmaking globally. It was developed to support decisionmaking by integrating into unified metrics some of the key elements considered to be important. Although the methods developed in this field are valuable for examining the consequences of new healthcare interventions, the focus on CE ratios (e.g. cost per quality-adjusted life year [QALY]) has contributed to a "black box" syndrome, both at the clinical and policy levels[1,2] In addition, healthcare decisions need to be based on a wider set of considerations that are not part of the CE model such as current need, lack of treatment and disease severity [3-6].

A number of multicriteria models have emerged to support deliberation and assist consideration of the numerous factors implicated in healthcare decisionmaking [7-15]. Some elements of decisionmaking can be quantified, and multicriteria decision analysis (MCDA) provides a way to account for multiple streams of information [16]. MCDA is emerging as a tool that goes beyond cost-effectiveness by allowing integration of more elements, such as disease severity [16-18]. In addition, MCDA provides a mechanism that allows decisionmakers to gain insight into their priorities and values [19]. However, not all elements of decision are quantifiable (e.g., ethics, historical context) and may be difficult to incorporate into an MCDA model. Culyer [20] suggested a process that blends algorithmic (quantitative) and deliberative (non-quantitative) approaches. Such a comprehensive framework should allow explicit consideration of all elements of decision by a wide range of stakeholders [21] to provide accountability for reasonableness [22].

Another critical point is how to inform decisionmakers on those elements of decision, the goal of health technology assessment (HTA) activities--currently carried out by governmental agencies, public and private payers and manufactures around the world [5,23]. HTA is as useful as the data available to build it, highlighting the critical impact of clinical trial design, which is heavily used to assess efficacy, safety, patient reported outcomes and economic outcomes [4], and the transparent reporting of results [1]. To fulfill their roles, HTA producers should also inform socio-ethical dimensions of new interventions [24]. However, although ethical evaluation helps stakeholders realize the consequences of implementing a healthcare intervention at the micro (patient), meso (institution) and macro (society) levels [25], only 47% of the International Network of Agencies for Heath Technology Assessment (INAHTA) member organizations reported including ethics in their assessments [26].

A decisionmaking framework bridging HTA with MCDA was proposed [27] that provided a pragmatic link between HTA and healthcare policy and clinical decisionmaking. In a proof-of-concept study, the preliminary framework was applied to 10 drugs and tested by 13 Canadian stakeholders during a panel session (submitted manuscript). In the current study, a complex case was tested to further explore the non-quantifiable elements of decision, to develop a comprehensive framework supporting consideration of all elements of decision, and to explore the validity of this approach. The use of growth hormone (GH) to treat patients with Turner syndrome (TS) was selected because of the complexity of the considerations surrounding expensive hormone injections over long periods of time to augment height in growth-delayed children affected by this syndrome. It was also chosen because it is an approved therapy and is used worldwide in developed countries for Turner syndrome, a genetic condition affecting between 1 in 2000 and 1 in 2500 live born females [28,29].

## Methods

### Study design

The Evidence and Value: Impact on DEcisionMaking (EVIDEM) framework includes a comprehensive set of standard components of healthcare decision and a process to consider each component, for which synthesized data is made available in a matrix format. Components that are quantifiable from a universal standpoint (defined as intrinsic value components) are structured into an MCDA matrix (the MCDA Value Matrix or VM), which includes 15 components usually considered in healthcare decisionmaking [27]. Other components of decisions, which are not quantifiable from a universal standpoint, i.e., related to the specific healthcare system or ethical considerations (defined as extrinsic value components [27]), were further explored, identified and structured into a tool (Extrinsic Value Tool - see below).

**Figure 1:**
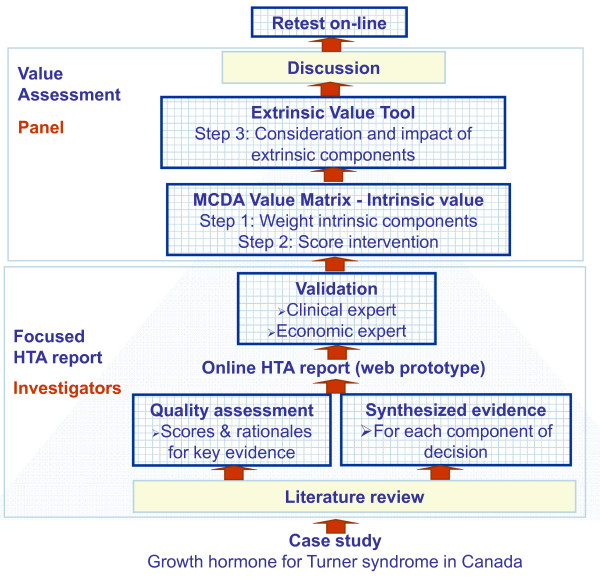
**Study plan**.

A synthesized HTA report on growth hormone for Turner syndrome tailored to each component of decision was prepared and validated by experts (Figure 1). A report on the quality of available evidence was generated using instruments for each type of evidence (Quality Matrix). A panel of stakeholders estimated the intrinsic value of growth hormone for Turner syndrome in Canada by assigning weights and scores. The impact of non-quantifiable (extrinsic) components of decision on value was then evaluated. The validity of the approach was explored by test-retest, discussion and survey.

### Development of the Extrinsic Value Tool

Non-quantifiable components of decision such as ethical and system-related considerations were identified based on:

• extensive review of the literature and decisionmaking processes;

• discussions with decisionmaking bodies and stakeholders during workshops presenting the EVIDEM framework; and

• discussions on extrinsic components of decision during the proof-of-concept study with the 13 Canadian panelists involved (policy decisionmakers, clinicians [specialists and general practitioners], nurses, pharmacists, health economists/epidemiologists) who evaluated 10 medicines using the EVIDEM framework (submitted manuscript).

Six components were thus defined: three components defining an ethical framework and three healthcare system-related components (Table 1). Components were organized into a tool that asks evaluators whether each component should be considered, and if so, would impact positively or negatively on the value of the intervention.

**Table 1:** HTA report with validated data for each component of decision of the framework (highly synthesized version)

Overview				
**Disease**: Turner syndrome (TS) **Intervention**: growth hormone (GH) **Setting**: Canada		**Drug class**: Polypeptide hormone **Indication**: treatment of short stature in girls with Turner Syndrome **Administration**: subcutaneous injection 3 to 7 days a week **Intervention duration**: not established; initiate treatment as soon as growth failure demonstrated until satisfactory height reached (in Canadian RCT, 6 years of treatment starting at 10 years) **Comparator(s)**: No treatment **Economic burden of illness**: No data available		

**Intrinsic value components** **(MCDA Value Matrix)**		**Highly synthesized information**	**Scoring of intervention**	
			
			**Minimum score (0)**	**Maximum score (3)**

**Quality of evidence**				

*Q1*	*Adherence to requirements of decisionmaking body*	*Not applicable for case study*	*Low adherence*	*High adherence*

Q2	Completeness and consistency of reporting evidence	**Epidemiology: **limited statistical information; **Clinical data: **limited reporting of AEs; **PRO: i**ncomplete reporting of questionnaire dimensions; **Economic evaluation: **some model features unclear; **Budget impact: **no sensitivity analysis reported	Many gaps/inconsistent	Complete and consistent

Q3	Relevance and validity of evidence	**Epidemiology: **study in one Canadian hospital with small sample size; **Clinical data: **uncertainty on final height gain, high attrition rate in key RCT; **PRO: **interim analysis of a subset of participants to a non blinded RCT; **Economic evaluation: **questionable outcome -cost per cm of final height, no adverse events costs, weak utility data; **Budget impact: **assuming all Canadian girls treated based on prevalence data	Low relevance/validity	High relevance/validity

**Disease impact**				

D1	Disease severity	**Female-specific genetic disorder **characterized by short stature, cardiovascular defects, absence of puberty, infertility, increased risk of diabetes, defects in visuo-spatial organization and nonverbal problem-solving, and decreased life expectancy	Not severe	Very severe

D2	Size of population	**Prevalence**: 40/100,000 female adults	Very rare disease	Common disease

**Intervention**				

I1	Clinical guidelines	**International guidelines (**no Canadian guidelines): Consider GH treatment as soon as growth failure is demonstrated and potential risks/benefits have been discussed with patient/family. Treat until satisfactory height is reached	No recommendation	Strong recommendation

I2	Comparative interventions limitations	There is no other therapeutic intervention indicated to treat short stature in Turner syndrome	No or very minor limitations	Major limitations

I3	Improvement of efficacy/effectiveness	**4 placebo controlled RCTs **(2-year (toddlers) to 11-year treatments; N = 42 to 104, 1 in Canada, 3 in USA): Final height of treated patients = 147 cm to 150 cm (excluding toddlers); difference with untreated = 7 cm **Observational controlled studies **(2-year to 8-year treatments, N = 26 to 123, 1 in Germany, 1 in Greece, 1 in Israel, 3 in Italy): Final height of treated patients = 148 cm to 151 cm; difference with controls = 2.1 to 6.8 cm	Lower than comparators	Major improvement

I4	Improvement of safety & tolerability	**Common AEs (from RCTs -frequency at least twice of placebo)**: Surgeries (50%), ear problems (6% to 47%), joint (13.5%) and respiratory (11%) disorders, sinusitis (18.9%) **Serious AEs (from registries, no control data): **Intracranial hypertension (0.2%), slipped capital femoral epiphysis (0.2 - 03.%), scoliosis (0.7%), pancreatitis (0.1%), diabetes mellitus (0.2 to 0.3%), cardiac/aortic events (0.3%), malignancies (0.2%) **Warnings: **Scoliosis, slipped capital femoral epiphysis, intracranial hypertension, ear disorders, cardiovascular disorders, autoimmune thyroid disease, insulin resistance	Lower than comparators	Major improvement

I5	Improvement of patient reported outcomes	Inconclusive data: **1 RCT **(2-year treatment data, N = 28, Canada): higher rating on questionnaire by GH treated patients versus untreated for some domains but not for others **2 observational studies**: no significant differences on SF-36 dimensions in one study (5-year treatment, N = 568, France) and significant differences in another (7-year treatment N = 29, Holland); other questionnaires, non significant differences **Convenience**: Subcutaneous injection 3 days a week or daily	Worse patient reported outcomes than comparators presented	Major improvement

I6	Public health interest	No data on **risk reduction **with GH treatment	No risk reduction	Major risk reduction

I7	Type of medical service	**Goal of treatment**: promote growth and improve psychosocial wellbeing (height gain 7 cm, patient reported outcomes data limited & inconclusive)	Minor service	Major service *(e.g. cure)*

**Economics**				

E1	Budget impact on health plan	**Average annual cost of drug per patient: **CAN$28,525 **Annual impact for Canadian public drug plans**: $11.3 million (coverage for all 396 Canadian patients)	Substantial additional expenditures	Substantial savings

E2	Cost-effectiveness of intervention	**Incremental cost per additional centimeter in final height**: $23,630 (discounted at 5%); **Incremental cost per QALY gained **$243,087 (discounted at 5%)	Not cost-effective	Highly cost-effective

E3	Impact on other spending	**Incremental cost per patient**: $1,166 (includes training by nurse, outpatient visits & X rays over 6 years - excludes drug cost, see E1)	Substantial additional spending	Substantial savings

**Extrinsic value components** **(Extrinsic Value Tool)**		**Highly synthesized information**	**Should this be considered? Would it impact positively or negatively on value of intervention?**	

Ethical framework*				

	Goals of healthcare - utility*	**Goal of healthcare **is to maintain normal functioning which may be impacted by very short stature. **Goals of GH treatment **are to promote growth and improve psychosocial adaptation of individual with short stature. However, psychosocial functioning of individuals with short stature is largely indistinguishable from their peers.		

	Opportunity costs- efficiency*	Considering maximizing impact on health for a given level of resources at: **Patient level**: resources allocated to GH treatment may be more beneficial if allocated to other interventions such as psychological support to cope with condition overall (not just short stature). **Society level: **Significant cost/person but small population.		

	Population priority & access - fairness*	**Prioritize worst off**: applicable to patients with Turner Syndrome but maybe not to the short stature part of the disease; daily lot probably not improved with daily injections for several years, but maybe as adult with less short stature than without treatment. **Treat like cases similarly**: should we treat differently short stature due to disease or due to genes? **Access to care/treatment**: easier in big cities where specialists are available		

**Other components**				

	System capacity and appropriate use of intervention	Optimal age for initiation of treatment has not been established. Appropriate follow up requires the intervention of skilled healthcare professionals In Canada, any physician can prescribed GH; some of the provinces that reimburse GH require it is prescribed by an endocrinologist.		

	Stakeholder pressures	Pressure from parents, from clinicians, industry?		

	Political/historical context	Societal pressure on short stature?		

	Other components?			

*Ethical framework based on three principles; when conflicting principles, clearly identify trade-offs and legitimate decision by engaging a broad range of stakeholders & explaining decision; legitimizing decision is key to provide accountability for reasonableness

An ethical framework, combined with involvement of all relevant stakeholders, are both critical elements in the legitimization of healthcare decisions [30,31] and they provide accountability for reasonableness [22,32,33]. Although some ethical aspects are included in the MCDA VM (e.g., the scale gives a higher value to treatments that target severe disease compared to those that target diseases that are not as severe), a more complete ethical framework was integrated into the Extrinsic Value Tool to make sure that additional ethical principles are explicitly considered. Standard ethical principles of a) utility, b) efficiency and c) fairness [34] were combined with considerations of a) the goal of healthcare, b) the opportunity costs, and c) the population priorities and access to healthcare, respectively. These three principles are often conflicting and the tool allows identification of potential trade-offs.

Three system-related components were included in the Extrinsic Value Tool, referring to non-scientific evidence defined by Lomas [21]. System and organizational capacity [8,21] and capacity to ensure appropriate use are critical contextual elements. Lobbying from various groups is often part of the whole mechanism leading to healthcare decisions [21] and should be made explicit to ensure that all interests at stake are known by the decisionmakers [35]. This also includes the interest of the decisionmakers themselves [26]. Political priorities and historical context, including habits, traditions, and precedence [21,36], may also affect the value of the intervention under scrutiny.

Although these six components are not quantifiable from a universal viewpoint and are thus considered qualitatively in the framework, some may become so in specific settings, providing that there is agreement on the low and high ends of the scale and that data can be gathered to operationalize them.

### Health technology assessment report

Synthesized evidence about growth hormone treatment for patients with Turner syndrome in Canada was prepared following the EVIDEM methodology [27]. An extensive analysis of the literature was performed to identify most relevant available data (i.e., data obtained in Canadian population, with comparative data for no treatment/placebo) supplemented by key studies in other settings. Databases and sources searched included PubMed, Centers for Review and Dissemination, Cochrane, trial registries, Disease Association web sites (Canadian Turner syndrome society; Turner Syndrome Society of United States; Eunice Kennedy Shriver National Institute of Child Health and Human Development, American Academy of Pediatrics; and Turner Syndrome society in France, UK, and Australia), websites of the Agency for Healthcare Research and Quality (AHRQ), the National Institute for Clinical Excellence (NICE), the Canadian Agency for Drugs and Technologies in Health (CADTH), and the World Health Organization (WHO), completed by hand searching of bibliographies. Search terms included: Turner syndrome, growth hormone/GH/rhGH/somatropin, quality of life/QoL/HRQoL, epidemiol*/prevalence/incidence, mortality, guideline/recommendation/clinical practice, patient reported outcome*/PRO, cost*, econom*, productivity, ethic*.

For clinical evidence, randomized controlled trials with complete comparative data on final or adult height (considered most important primary outcome [37]) were included. Although it did not report adult height, a randomized controlled clinical trial with 2-year old patients was also included to inform potential changes in clinical practice in treating very young patients. Summary data from observational studies was included if they reported final height, based on the assertion that considering both types of evidence better informs decisionmaking [38]. Safety data was obtained from registries, clinical trials and product monographs. Patient reported outcomes (PRO), epidemiological and economic data in Canada was supplemented by data from other countries. Canadian clinical guidelines were not available and guidelines from US were used. Data thus selected was synthesized for each component of the MCDA VM to inform scoring.

The quality of evidence found was assessed using the Quality Matrix (QM) instruments [27] for five types of evidence (clinical, patient reported outcomes, epidemiological, economic and budget impact analysis) and for two criteria of quality ("completeness and consistency of reporting" and "relevance and validity of evidence"). For each type of evidence, studies most relevant to the Canadian setting were assessed. Due to the subjective nature of these types of assessments [39], transparent reporting of a critical analysis of each study was combined with a three-step process to reach a consensus. First, a trained investigator reviewed the study, provided comments for each dimension of the QM instruments, a score and the rationale for that score for each study (or group of studies, e.g., clinical trials). All evaluations were then reviewed by a second trained investigator and validated by an expert in the field (Figure 1).

To explore the extrinsic value of growth hormone for patients with Turner syndrome, a review of the literature on the ethical, psychosocial and contextual aspects of growth hormone treatment was performed. Concepts and information were categorized and synthesized using the Extrinsic Value Tool.

The HTA report thus generated was programmed into an interactive web based prototype using Tikiwiki v2.2 The prototype allowed experts performing validation to access the HTA report online as well as full text source documents, and to enter feedback on the synthesized data, critical analysis of evidence, and quality scores. Clinical, PRO, and epidemiological data was validated by a clinician with extensive expertise in Turner syndrome. Economic data was validated by a health economist.

### Panel

The panel was designed to include relevant stakeholders, with a focus on experts in the disease to explore relationships between policy and clinical decisionmaking. Stakeholders were contacted by email with an invitation letter describing the project, were offered identical minimal honoraria, and their expenses were covered. The panel was composed of:

◦ 4 academic pediatric endocrinologists with extensive clinical experience with trial design and with patients with Turner syndrome

◦ 1 ethicist who was also a pediatrician endocrinologist

◦ 1 nurse with extensive clinical experience with Turner syndrome

◦ 1 Turner syndrome patient/patient group representative

◦ 2 health economists/epidemiologists who had exposure in health policy decisionmaking.

### Value of growth hormone for Turner syndrome

The web-based prototype was used by the nine panelists to read the HTA report prior to the panel session. Each panelist then applied the framework during the panel session (test) to assess the value of growth hormone for patients with Turner syndrome. This was repeated online at least two weeks after the panel session (re-test) (Figure 1). Intrinsic value was assessed using the MCDA VM (steps 1 and 2) and extrinsic value using the Extrinsic Value Tool (step 3).

◦ Intrinsic value (estimated by combining weights and scores):

◦ Step 1: Weighting of MCDA intrinsic value components independently of intervention and from a societal perspective; a scale of 1 to 5 was used.

◦ Step 2: Scoring of MCDA intrinsic value components for the intervention using the synthesized data reported in the MCDA VM and a scoring scale of 0 to 3 with defined anchors and scoring examples; the MCDA VM included features to collect feedback on the synthesized data and the evaluating process, and to specify whether a low score was due to data limitations.

◦ Extrinsic value

◦ Step 3: Considering extrinsic value components and their impact on the value of growth hormone for patients with Turner syndrome using synthesized data.

Feedback was also collected during a discussion period during the day of the panel and from a questionnaire administered after the panel session.

### Data collection and statistical analyses

For the panel evaluation (test), weights, scores and consideration of extrinsic components were obtained on the hardcopy documents distributed to panelists and entered in Excel software. Data entered on-line by panelists (retest) was recorded in a MySQL database and transferred to the Excel software, which was then used to perform statistical analyses.

The estimated intrinsic value of growth hormone for Turner syndrome was obtained by applying an MCDA linear additive model combining normalized weights and scores for all components of the MCDA VM [27]. Mean, standard deviation (SD), minimum and maximum values were calculated. MCDA value estimates from one evaluator differed by more than 50% between test (2.0 or 75%) and retest (1.15 or 38%), indicating systematic error, and was excluded from statistical analyses.

Agreement between test and retest data was analyzed by calculating intra-rater correlation coefficients (ICCs) for weights, scores and MCDA value estimates. Two types of ICCs were calculated following Shrout and Fleiss (1979) [40] methods and classification: the ICC (3,1) which is based on a two-way mixed analysis of variance (ANOVA) model (general effects of the test and the retest were assumed to be fixed); and the ICC (1,1), which is based on a one-way ANOVA model and assumes that test and retest data do not differ in a systematic way and are therefore interchangeable. In addition, the proportion of data pairs that did not differ between test and retest, differed by 1 point, and by 2 points, was calculated for weights and scores.

Inter-rater correlation coefficients were not calculated since the tool is designed to capture personal values and perspectives, which are expected to vary across individuals.

## Results

### Health technology assessment report

The HTA report summarized current knowledge on growth hormone for patients with Turner syndrome within the Canadian context and was validated by experts. Data was organized to directly feed into the MCDA VM and the Extrinsic Value Tool to provide, in a practical manner, the data that is necessary to consider each element of decision. A highly synthesized version of the data presented to panelists is reported in Table 1 (details, referencing, and access to sources are available on the collaborative registry at http://www.evidem.org/evidem-collaborative.php). Because there is no other therapeutic intervention indicated to treat short stature in Turner syndrome, no treatment was used as the standard comparative treatment option.

Growth hormone is the only available intervention indicated for the treatment of short stature in girls with Turner syndrome, a rare (1 in 2000) genetic disorder characterized by reduced life expectancy, absence of puberty, cardiovascular defects and short stature (about 20 cm lower that mean adult height of North American women). Treatment requires daily injections over several years but optimal duration of treatment and age of initiation have not been established. Compared to no treatment, randomized controlled trials and observational studies report an average height gain of 7 cm, and 2 to 7 cm, respectively, for a final height of about 150 cm. Growth hormone carries many warnings and its safety profile in Turner syndrome patients is characterized by increases in middle ear problems & related surgeries (vs no treatment, randomized controlled trial) as well as very rare but serious adverse events reported in registries. The impact it has on the patient quality of life is inconclusive with limited data. Thus beyond increasing height, it is unknown whether growth hormone provides long-term quality of life benefits, a problem common to HTA of many drug therapies. The annual drug cost per patient is about CAN$29,000, with other costs estimated at about $1,200 per year. The annual budget impact on drug plans in Canada is estimated to be $11.3 million and its cost per QALY ranges from $56,000 (not discounted) to $243,000 (discounted).

Other aspects of the decision were identified and reported in the Extrinsic Value Tool including the (mis)alignment of growth hormone with the goal of healthcare to maintain normal functioning, optimal allocation of resources at patient and society level, fairness in treating short stature, potential inappropriate use given limited guidelines on optimal treatment and potential cultural and stakeholder pressures on short stature.

### Value of growth hormone for Turner syndrome

**Figure 2:**
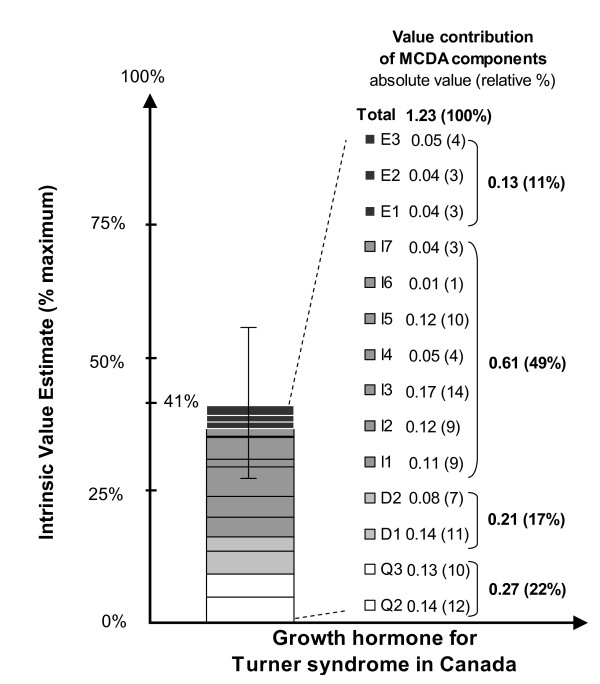
**Intrinsic value estimate for intervention on the MCDA Value Matrix scale and value contribution of each component**. *For an intervention to achieve close to 100% on this scale, it would have to cure a severe endemic disease, demonstrate a major improvement in safety, efficacy and PRO compared to limited existing approaches, and result in major healthcare savings. Conversely, an intervention that scores low would be for a rare disease that is not severe, with minimal improvement in efficacy over existing alternatives, with major safety and PRO issues and resulting in major increases in healthcare spending.

**Figure 3:**
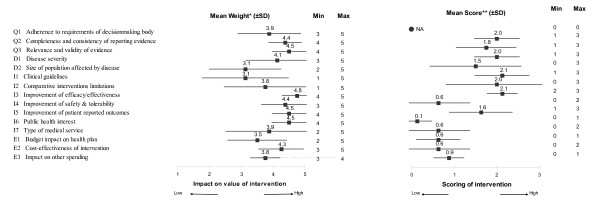
**Weights for MCDA Value Matrix components and scores for growth hormone for Turner syndrome in Canada (average data from eight panelists)**. *A five point weighting scale was used with 1 lowest and 5 highest weight. **A short four point scoring scale was used with 0 lowest (to account for component that would not bring any value) and 3 highest score.

The mean intrinsic MCDA value estimate of growth hormone for patients with Turner syndrome was 1.23 (41% of maximum value), ranging from 0.79 (26%) to 1.61 (54%) among panelists (Figure 2). This was obtained by a linear combination of normalized weights and scores, for which large variations between panelists were observed (Figure 3). The intrinsic MCDA estimate was a reflection of:

1- Personal values & perspective (weights) of panelists regarding the relative importance of each component of decision; at the panel level, "Improvement of efficacy" was identified as the most important component (4.8 ± 0.5), and "Size of population" and "Clinical guidelines" as the least important (3.1 ± 1.1 and 3.1 ± 1.4, respectively; Figure 3)

2- Comprehensive performance (scores) of the intervention for a range of quantifiable components (Figure 3)

Contribution of the cluster of components categorized as "Intervention" to the MCDA estimate was the most important (50%), while the "Economics" cluster contributed least (11%) (Figure 3). At the component level, the main contributors were "Improvement of efficacy (I3; 14% of total value)", followed by disease severity (D1; 11%), quality of evidence (Q2 and Q3; 11% & 10%) and limitations of comparative interventions (10%) (Figure 3).

This MCDA value estimate laid the groundwork for ethical and healthcare system related considerations (Table 2). The impact of these on the value of the intervention was mixed and sometimes conflicting, highlighting the importance of explicitly considering such components as part of the entire process of decisionmaking.

**Table 2:** Extrinsic value tool: component definitions and panelists' considerations on the value of growth hormone for Turner syndrome

Extrinsic value components	Definition	Panelists' considerations
Ethical framework*		
Goals of healthcare - utility*	Goal of healthcare is to maintain normal functioning. Such consideration is aligned with the principle of utility, which considers the act to produce the greatest good or "greatest benefits for the greatest number"	**Considered**: Panelists reported that the goal of healthcare (i.e., addressing medical issues rather than social issues) should be considered, and that height is not entirely a social issue, but also a medical issue for very short patients. **Impact**: Therefore for very small patients, there is a medical utility in facilitating normal functioning (reaching car pedals, kitchen cabinets etc), which would impact positively on the intrinsic value of the intervention. On the other hand, weak evidence linking improvement in minor short stature with personal gain would have a negative impact on value.
Opportunity costs- efficiency*	Opportunity costs include resources or existing interventions that may be forgone if intervention under scrutiny is used/reimbursed. Such consideration is aligned with the principle of efficiency, which considers maximizing impact on health for a given level of resources (efficiency can be considered at the patient level and at the society level)	**Considered**: Panelists indicated that this should be considered to capture the opinions of stakeholders **Impact**: would have a negative impact on value, more value might be derived from psychosocial support. **Comment**: Resources are often allocated to measurable outcomes (e.g., height) rather than softer outcomes such as psychosocial benefits.
Population priority & access - fairness*	Priorities for specific groups of patients are defined by societies/decisionmakers and reflect their moral values. Such considerations are aligned with the principle of fairness, which considers treating like cases alike and different cases differently and often gives priority to those who are worst-off (theory of justice)	**Considered**: Panelists indicated that this should be considered **Impact**: mixed impact - negative impact related to the concept of treating like cases similarly (e.g., short stature due to other diseases) as it dilutes the importance of TS patients relative to other groups **Comment**: should not discriminate against rare diseases; there should be public debate on priorities
**Other components**		
System capacity and appropriate use of intervention	The capacity of healthcare system to implement the intervention and to ensure its appropriate use depends on its infrastructure, organization, skills, legislation, barriers and risks of inappropriate use. Such considerations include mapping current systems and estimating whether the use of the intervention under scrutiny requires additional capacities (note: if available, economic estimate would be included in the economic component E3 of the MCDA Value Matrix)	**Considered**: some panelists indicated that it should be considered while others indicated there was no potential for inappropriate use **Impact**: for those who indicated it should be considered, it would have a negative impact on value **Comment**: although there is no risk of misdiagnosis (genetic testing), because guidelines are not clear on age initiation, there is a risk of having all toddlers initiated on treatment, which was considered as inappropriate use. Misuse of growth hormone is possible (gaining height for no medical reason) and there are no mechanisms in place for surveillance of inappropriate use.
Stakeholder pressures	Pressures from groups of stakeholders are often part of the context surrounding healthcare interventions. Such considerations include being aware of pressures and interests at stake and how they may affect values of decisionmakers	**Considered**: some panelists indicated that it should be considered while others reported that it should not be taken into account **Impact**: for those who indicated it should be considered, it would have a negative impact on value. **Comment**: Lobby groups are effective at reaching and impacting decisionmakers
Political/historical context	Political/historical context may influence the value of an intervention in consideration of specific political situations and priorities as well as habits, traditions and precedence	**Considered**: Panelists indicated that this should be considered **Impact**: none reported **Comment**: This includes the political will to demonstrate fairness to rare disorders as well as universal access to care (guaranteed by the Canadian healthcare system) to satisfy entitlement felt by affected families. Budgetary context (i.e., recession, balanced budget or surplus) affects decisions.
Other components	Components that are not already captured in the standard set proposed	

*Ethical framework based on three principles; when conflicting principles, clearly identify trade-offs and legitimize decision by engaging a broad range of stakeholders & explaining decision; legitimizing decision is key to provide accountability for reasonableness

**Table 3:** Agreement at the individual level between test-retest for weights, scores and MCDA estimates obtained with the MCDA Value Matrix

	Weights	Scores	MCDA Estimates
Number of test-retest pairs	120*	112^†^	8
Mean of test data	4.03	1.33	1.23
Mean of retest data	3.74	1.28	1.20
ICC (3,1)	0.578	0.681	0.656
ICC (1,1)	0.546	0.682	0.687
Proportion of pairs with no test-retest difference (%)	50.8	65.2	NA
Proportion of pairs with test-retest difference of 1 point (%)	39.2	28.6	NA
Proportion of pairs with test-retest difference of 2 points (%)	10.0	6.3	NA

*8 evaluators × 15 components; ^†^8 evaluators × 14 components (component Q1 was not scored for case study)

NA: Not applicable

ICC: intra-class correlation coefficient, defined according to Shrout and Fleiss (1979) [40]

In reviewing the synthesized data of the MCDA VM, a discussion was sparked about what comprises a meaningful outcome for the patient (what is the value of a statistical difference of 7 cm?), highlighting the importance of the original research question. Limited data on quality of life benefits and failure to compare growth hormone treatment with psychosocial support or other strategies for wellbeing was also noted, as was absence of long term comparative data for a treatment with a lifetime impact. Cost-effectiveness data--especially wide variation between discounted and undiscounted data results--caused frustration among panelists regarding the real significance of these metrics, especially since discounting disadvantages children, which was seen as inequitable. Beyond its impact on drug costs, there was little short term data regarding the economic impact of growth hormone on other healthcare resources, and complete absence of long-term data, all of which severely limited interpretation and assessment of component E3 (Impact on other spending).

### Exploratory validation of approach

When surveyed whether each component of decision of the framework (Intrinsic and Extrinsic Value Components) should be considered in the decisionmaking process, panelists indicated that they would consider most of them except for "Stakeholders pressures" (33% of panelists would not consider this component), "Clinical guidelines" (25%), "Adherence to requirements of decisionmaking body" (13%) and "Type of medical service" (11%).

Applying the MCDA VM during the panel (*test*) and then re-applying it individually on-line (*retest*) yielded mean MCDA value estimates of 1.23 for the test and 1.20 for the retest (Table 3). The ICC (3,1) was 0.656 and the ICC (1,1) 0.687 indicating fair to good reproducibility. When the data from the outlier was included, ICC (1,1) dropped to 0.367 and ICC (3,1) to 0.371. Data below is excluding the outlier.

Weights tended to show lower test-retest agreement than scores, with ICC (3,1) of 0.578 and 0.681 for weights and scores, respectively, indicating that they were to a larger degree responsible for test-retest disagreements in MCDA value estimates than changes in scores.

Approximately half of the 120 weights (50.8%) were identical between test and retest, 39.2% differed by 1 point (on a scale of 1 to 5), and 10.0% differed by 2 points. Cluster D (comprised of components *Disease severity *and *Size of population affected by the disease*) recorded the greatest disagreement between test and retest weights (only 37.5% were identical) of the four clusters of the VM (i.e., Q: Quality of data, D: disease description, I: intervention characteristics, E: economics).

With respect to test-retest comparison of scores, 65.2% were identical, 28.6% differed by 1 point (on a scale of 0 to 3), and 6.3% differed by 2 points. Cluster E (comprised of the 3 components *Budget impact on health plan*, *Cost-effectiveness of intervention*, and *Impact on other spending*) recorded the greatest variation in scores between test and retest (58.3% of scores were identical; 12.5% differed by 2 points) of the four clusters. The components *Cost-effectiveness of intervention *and *Impact on other spending*, both based on cost-effectiveness analysis, were largely responsible for the greater disagreement in scores for Cluster E, while scores for the component *Budget impact on health plan *showed little disagreement between test and retest.

## Discussion

The current debate on transparency and legitimacy of healthcare decisionmaking [41,42] calls for a more systematized approach to evidence review and decisionmaking. In particular, ethical and colloquial (e.g., not based on scientific evidence) considerations are inherent to the thought process underlying healthcare decisionmaking [21,43] but are often not explicitly acknowledged or discussed, let alone communicated to other stakeholders. The EVIDEM framework proposes a comprehensive set of components of decision to consider in making healthcare choices, and transparent access to available data on which these considerations are based. This approach is envisioned to help identify the issues at play and increase legitimacy and transparency of decisions.

Testing the EVIDEM framework using the complex case of growth hormone for patients with Turner syndrome allowed development of a complementary tool (Extrinsic Value Tool), thus expanding the comprehensive nature of the framework. Applying the framework to this case clearly exposed limitations of the cost-effectiveness paradigm and highlighted the importance of systematically considering all relevant aspects of an intervention to best reach an informed decision. It also revealed gaps in available data and a need to better align research questions with data needs. Exploratory validation of the approach provided support for the inclusion of most framework components and showed good agreement of MCDA value estimates at the individual level (test-retest) but large variations across panelists reflecting different perspectives and personal values.

This case study exemplifies how MCDA can provide a means to integrate a wider range of components into the decisionmaking process [16] and help consider each of them explicitly. The use of growth hormone is complex and raises many questions, including far reaching issues such as social perception of short stature (What is short stature? Why is it an issue? What is the value of an incremental gain in stature?) In addition, consideration of the ethical components of the Extrinsic Value Tool brought up questions of fairness towards Turner syndrome patients versus other individuals with short stature [33,44]. Decisionmaking that solely relies on the cost per QALY ignores these issues, leaving them at best to ad hoc discussions. The clear inadequacy of the available utility data and the great difference between discounted and non-discounted incremental cost-effectiveness ratios (because high costs are incurred during childhood, but benefits accumulate over adult life-time) observed for this case study cast additional doubt upon reliance on the cost per QALY measure and highlight the usefulness of a more comprehensive approach.

In a first step, applying the framework allows eliciting the personal values of the committee/panel members (weighting). Variations across individuals are expected and were observed in the case study reflecting different perspectives and potential bias. Of note, panelist weights were lowest for "Clinical guidelines", suggesting limited impact of these, perhaps related to the current debate surrounding guideline limitations [45].

In a second step, combining panelist weights and scores for the intervention, produces an MCDA value estimate--a panel-specific comprehensive measure integrating relative value (i.e., comparative efficacy, safety, patient reported outcomes, impact on treatment budget and other spending, cost-effectiveness), absolute value (i.e., severity of disease, size of population affected, types of benefit at population and patient level, quality of evidence), as well as clinical guidelines and the limitations of alternative interventions. In this case study, the MCDA estimate was 41% of maximum value, with major drivers including "Improvement of efficacy" (14% of total MCDA estimate), "Disease severity" (11%) and "Quality of evidence" (22%, for Q2 and Q3 combined). Although not directly comparable due to different panel compositions, this MCDA estimate is at the lower range (42% - 64%) of estimates obtained during the proof-of-concept study for 10 medicines approved in Canada (submitted manuscript). Further research is needed to position MCDA estimates within a frame of reference.

The process of considering the quantifiable components of decision lays the groundwork for the third step in which qualitative ethical and healthcare system related factors are being considered. The MCDA estimate is not intended to be used in a formulaic approach [16], but rather as an attempt to tease out all relevant quantifiable components and to then consider the impact of other ethical and contextual elements influencing overall value. In this case study, issues identified included the medical utility of height gain (Utility), provision of psychosocial support as an alternative (Efficiency), policy towards rare diseases with respect to discrimination and priority setting (Fairness, Political context) and potential misuse in toddlers (System capacity/appropriate use). Although these issues had mixed effects on the perceived value of the intervention under scrutiny, panelists noted that the process helps ensure awareness of all historical, political, system-related and ethical elements that may impact the decision. Overall, the framework encouraged a better analysis of the issues that troubled panelists subconsciously, rendering them more explicit in this assessment.

Exploring the validity of the framework revealed that panelists would consider most of its components to assess a healthcare intervention, thus providing some support for the current set of components. Although validation in terms of agreement across panelists was not possible since the tool is designed to capture differences of personal values, exploration of intra-rater agreement between weights and scores obtained during test and retest revealed a fair to good agreement at the individual level (ICC = 0.6). Weights were, to a larger degree than scores, responsible for test-retest disagreement in value estimates, suggesting that panelists were still wrestling with their own values, or interpreted the components of the MCDA VM differently. Alternatively, this may have arisen because the panel was not yet comfortable enough with the process; this latter hypothesis is testable by examining individual test-retest data over time. For weights, largest variations were observed for disease-related components suggesting some difficulty in deciding on the importance of disease severity (D1) and size of population (D2). It may also reflect difficulty in positioning Turner syndrome on a scale encompassing all diseases, since specialists typically focus on their practice and tend to rank diseases and interventions within the range of patients they see [25]. For scores, the largest degree of test-retest disagreement at the individual level was observed for cost-effectiveness-based components, highlighting the difficulties in making sense of CE ratios and frustration associated with the systematic negative effect of discounting on cost/QALY ratios for interventions for children. Although CE ratios were included in the framework to make a connection with the current predominant model, this may not be necessary and separately considering definite expenditures (e.g., drugs costs - E1) and potential savings (e.g., hospital stay - E3) may be more informative and better aligned with the natural thought process underlying decisionmaking.

Discussing amongst a range of stakeholders all the components of decisionmaking on the basis of a structured HTA report that presents the available evidence (in its broad sense, i.e., both scientific and colloquial) for each component of decision was seen by the panelists as a useful means of revealing data gaps and identifying research questions. This can lead to improved study design and hopefully ensure better alignment of data generation with data needs at the micro, meso and macro levels. In this case, the process revealed the need for defining truly relevant outcome measures. From the patient perspective, height increase was seen as useful if it could permit normal functioning by bringing the patient from very small to small, highlighting the importance of patient involvement in sound decisionmaking [46-48]. Comparative data concerning alternative strategies to improve adaptation of individuals with short stature (e.g., psychological support) was also limited, but seen as important to assess the value of growth hormone, in line with previous studies [49,50].

### Limitations

Results of this case study should be considered in light of limitations. As stated in the introduction, any HTA is only as useful as the data available to build it, but it is also dependent on the care taken in collecting and extracting all appropriate data for analysis. While this is a laborious process, it is hoped that the availability of an open-source data repository will lead to time savings, since the same data is currently gathered independently by many individuals generating HTA. Integrating both the data and the framework in one panel session was challenging for some panelists. Although the panelists involved in the proof-of-concept study (submitted manuscript), who each evaluated several interventions using the MCDA VM, felt that the process became fairly clear after a couple of applications, a simplified version of data provided was developed for further testing (a 'lite' version).

Although variations across panelists in weights and, to a lesser extent, in scores are expected due to diverse perspectives and values, they may also be due to different data interpretation or misunderstanding (of data or of process), highlighting the importance of good coordination and communication to integrate the deliberative process [20] where there are several thought processes and perspectives at play (which can be captured at the individual level with the framework). Value assessments and considerations are those of a small group of stakeholders, which is often the case in the policy decisionmaking process.

We used a direct method for weight elicitation, which may not capture implicit or unconscious thoughts or preferences [51]. However, one of the objectives of the EVIDEM framework is to raise awareness of the components underlying decisionmaking. It is our hope that such process will make streams of thought more explicit and easier to communicate among stakeholders. Finally, this study was not designed to directly compare EVIDEM to other approaches used by decisionmaking bodies, such as cost-utility thresholds, and field studies comparing the EVIDEM approach to current decisionmaking processes are being developed.

## Conclusion

The EVIDEM framework is proposed as a step beyond the current cost-effectiveness model, combining efficient HTA, pragmatic MCDA, and an ethical framework to ensure systematic consideration of all components of decisionmaking and evidence available, and to provide a transparent record of how these elements have been used to reach a decision. It constitutes a concrete step towards addressing some of the elements identified as key to successful decisionmaking [42] and for accountability for reasonableness [22]. Systematically considering all pieces of the puzzle for healthcare interventions by all stakeholders leads to difficult questions, helps to position interventions across a wide range of options, including preventive and curative programs as advocated by Nord [3], and may enhance social responsibility and reduce bias.

The framework is primarily designed to stimulate and make more explicit the natural thinking process underlying decisionmaking and deliberation. Although decisionmaking contexts are diverse, we propose that the same comprehensive set of components can be considered in a broad range of circumstances because they can be easily adapted to user needs at both policy and clinical levels. HTA reports organized in the format of this framework to inform components deemed necessary can be used to stimulate reflection and deliberation. MCDA estimates can also be used to establish a ranking system that can encompass a broad range of healthcare interventions and is consistent with user priorities and values. The framework is applicable to decisions on healthcare interventions for which there are many comparators since it provides a pragmatic reporting of comparative effectiveness; this is currently being tested for pain management. The framework is also applicable for research planning, communication and knowledge translation; the last is currently being explored through a web-based open-access collaborative registry structuring data on healthcare interventions (high level synthesis, details and full text sources) using the framework components of decision. Further testing and validation is needed to build up MCDA approaches combined with pragmatic HTA in healthcare decisionmaking.

## Abbreviations

AEs: Adverse events; ANOVA: Analysis of variance; CE: Cost-effectiveness; EVIDEM: Evidence and Value: Impact on DEcisionMaking; GH: Growth hormone; HTA: Health technology assessment; ICC: Intra-rater correlation coefficients; INAHTA: International Network of Agencies for Heath Technology Assessment; MCDA: Multicriteria decision analysis; PRO: Patient reported outcomes; QALY: Quality-adjusted life year; QM: Quality Matrix; RCT: Randomized controlled trial; SD: Standard deviation; TS: Turner syndrome.

## Competing interests

The authors declare that they have no competing interests.

## Authors' contributions

MMG, DR and MW conceived the framework. MW and HK participated in data collection and drafting of the manuscript. JPG and CD reviewed and validated synthesized data. All authors read and approved the final manuscript.
